# Portable Automated Rapid Testing (PART) for auditory assessment: Validation in a young adult normal-hearing population

**DOI:** 10.1121/10.0002108

**Published:** 2020-10-06

**Authors:** E. Sebastian Lelo de Larrea-Mancera, Trevor Stavropoulos, Eric C. Hoover, David A. Eddins, Frederick J. Gallun, Aaron R. Seitz

**Affiliations:** 1 Psychology Department, University of California, Riverside, 900 University Avenue, Riverside, California 92521, USA; 2 Brain Game Center, University of California Riverside, 1201 University Avenue, Riverside California 92521, USA; 3 University of Maryland, College Park, Maryland 20742, USA; 4 University of South Florida, Tampa, Florida 33620, USA; 5 Oregon Health and Science University, Portland, Oregon 97239, USA

## Abstract

This study aims to determine the degree to which Portable Automated Rapid Testing (PART), a freely available program running on a tablet computer, is capable of reproducing standard laboratory results. Undergraduate students were assigned to one of three within-subject conditions that examined repeatability of performance on a battery of psychoacoustical tests of temporal fine structure processing, spectro-temporal amplitude modulation, and targets in competition. The repeatability condition examined test/retest with the same system, the headphones condition examined the effects of varying headphones (passive and active noise-attenuating), and the noise condition examined repeatability in the presence of recorded cafeteria noise. In general, performance on the test battery showed high repeatability, even across manipulated conditions, and was similar to that reported in the literature. These data serve as validation that suprathreshold psychoacoustical tests can be made accessible to run on consumer-grade hardware and perform in less controlled settings. This dataset also provides a distribution of thresholds that can be used as a normative baseline against which auditory dysfunction can be identified in future work.

## I. INTRODUCTION

The assessment of auditory function in modern clinical audiology was translated from the laboratory in the middle of the previous century (Carhart and Jerger, 1959; Hughson and Westlake, 1944) and has remained focused on using pure-tone audiograms to evaluate audibility and speech tests to assess the ability to detect particular acoustical cues in speech (see CHABA, 1988). These clinical assessments are targeted at the diagnosis of a hearing impairment based on audibility and on an approach to rehabilitation that is largely defined by its reliance upon amplification via hearing aids or cochlear implants. This focus on audibility and amplification has provided little incentive for clinical care to include the assessment and rehabilitation of suprathreshold auditory processing disabilities. As a result, there are very few tools and even fewer protocols available for the diagnosis and/or treatment of auditory difficulties that are not accompanied by losses of audibility. The diagnostic and rehabilitative approaches that do exist are regarded as specialized tools to be used by those clinicians who work with children or adults with suspected auditory processing disorders (APDs). There is a long history of clinicians and scientists using the term APD (e.g., Iliadou *et al.*, 2018); yet, some clinicians and researchers are uncomfortable with the term due to the potential overlap of APD with language and cognitive dysfunction (e.g., Moore, 2018). The perspective taken by this study is that regardless of the clinical status of APD, it is undeniably the case that tests of auditory perceptual abilities (e.g., Moore *et al.*, 2014; Eddins and Hall, 2010; Gallun *et al.*, 2013) have the potential to shed light on complaints of hearing difficulties that are only weakly predicted by the audiogram or performance on clinical speech tests (Hoover *et al.*, 2017; Eckert *et al.*, 2017; Souza *et al.*, 2018).

Clinically accessible tests of functional hearing are needed to better understand self-reported difficulties with auditory perception and poor performance on laboratory tests of auditory processing. These tests would need to be applied and validated across a population with diverse hearing abilities in order to clearly characterize which measures are most informative about the variety of hearing difficulties experienced by individual listeners or groups of listeners. Although a number of candidate tests have been developed and are relatively well studied in laboratory settings (e.g., Moore, 1987; Grose and Mamo, 2012; Bernstein *et al.*, 2013; Gallun *et al.*, 2014; Füllgrabe *et al.*, 2015; Jakien *et al.*, 2017; Hoover *et al.*, 2017; Hoover *et al.*, 2019), very few of these tests have been translated into standard clinical practice. Those tests that have been translated into the clinic are generally only used by audiologists with expertise in APDs because the testing often requires specialized equipment or setup and a calibrated audiometer. Even when the tests are built into the audiometer, many audiologists have not received adequate training to feel comfortable administering, scoring, and interpreting the tests.

Tests that have moved successfully from the laboratory to the clinic include the Staggered Spondaic Words test (SSW; Katz, 1962; Arnst, 1981), the Gaps in Noise test (GIN; Plomp, 1964; Green, 1971), the Masking Level Difference (MLD; Hirsh, 1948; Olsen *et al.*, 1976), the Dichotic Digits Test (DDT; Broadbent, 1958; Musiek, 1983), the Listening in Spatialized Noise test (LISN; Cameron and Dillon, 2007; Glyde *et al.*, 2013), the Frequency Patterns Test (FPT; Musiek and Pinheiro, 1987; Musiek, 1994), and the Dichotic Sentences Test (DST; Fifer *et al.*, 1983). In addition, the screening test for auditory processing (SCAN; Keith, 1995) is a battery of assessments that incorporates multiple auditory processing abilities. While these and other tests have been used successfully both in the laboratory and the clinic to identify auditory processing dysfunction (e.g., Gallun *et al.*, 2012; Gallun *et al.*, 2016; Hoover *et al.*, 2017), none of them are portable, automated, or rapid. They all require specialized equipment, such as an audiometer, and demand a trained audiologist to administer (most take at least 30 minutes) and score them by hand. The goal of this research project is to supplement these well-established tests with a low-cost, portable test system that could be used to administer a key set of basic auditory processing tests that is scored automatically and requires minimal clinical involvement. The assessments should each be rapid enough that clinicians and clinical researchers could tailor the length of the test battery to the time available. Moreover, portable automated rapid testing could play an essential role in gathering the datasets necessary to better characterize the auditory processing abilities and difficulties of individual listeners relative to the expected abilities of other listeners of a similar age with similar audiometric thresholds. Without this information, the clinician will continue to have difficulty appropriately identifying and remediating the auditory processing dysfunction they observe in their patients.

To address this gap, several state-of-the-art psychometric tests currently used in the laboratory to research central auditory processes have been translated into the application PART (Portable Automatic Rapid Testing) developed by the University of California Brain Game Center.^1^ PART can run both on mobile devices (e.g., iPad and iPhone, Apple Inc., Cupertino, CA; Android, Google, Mountain View, CA) and standard desktop computers (MacOS, Apple Inc., Cupertino, CA; Windows, Microsoft, Redmond, WA) and is currently freely available on the Apple App Store, the Google Play Store, and the Microsoft Store. PART has proven to be capable of accurately reproducing precise acoustic stimuli on an iPad with Sennheiser 280 Pro headphones (Sennheiser electronic GmbH and Co. KG, Wedemark, Germany) at output levels set by the built-in calibration routine (Gallun *et al.*, 2018).

The psychophysical test battery evaluated here was designed to reflect a description of the central auditory system, inspired by current research in psychoacoustics and auditory neuroscience (e.g., Stecker and Gallun, 2012; Bernstein *et al.*, 2013; Depireux *et al.*, 2001). This test battery is comprised of three sub-batteries, each with supporting evidence of clinical utility: temporal fine structure (TFS) processing, spectro-temporal amplitude modulation (AM), and targets in competition. These three groups of tests address different stages of auditory processing in the central nervous system that together mediate our ability to parse the auditory scene (Bronkhorst, 2015; Gallun and Best, 2020). We note that the test battery reported in this manuscript represents only a small subset of PART's functionality, and the PART platform facilitates a wide range of psychoacoustical tests.

TFS coding is assumed to rely upon the precision of phase-locking in populations of auditory nerve fibers responding to movements of the cochlear partition (Pfeiffer and Kim, 1975). The fine temporal information carried by the auditory nerve serves as the input to both the binaural system (see Stecker and Gallun, 2012) and the monaural pitch system (see Winter, 2005). Further refinement of this and other spectral and temporal information carried by the auditory nerve is responsible for the spectro-temporal modulation (STM) sensitivity observed in the inferior colliculus (Versnel *et al.*, 2009) and auditory cortex (Kowalski *et al.*, 1996). TFS sensitivity has been evaluated psychophysically using both monaural and binaural stimuli (Grose and Mamo, 2012; Gallun *et al.*, 2014; Hoover *et al.*, 2019). Neither the audiogram nor most conventional speech tests evaluate the detection of frequency modulation or use spatialization of auditory signals; yet, it has been found that TFS measures are a good predictor of speech understanding in competition (Füllgrabe *et al.*, 2015) and are suitable tests for age-related temporal processing variability (Grose and Mamo, 2012; Gallun *et al.*, 2014; Füllgrabe *et al.*, 2015). In this study, diotic frequency modulation was used to assess monaural TFS sensitivity, and dichotic frequency modulation was used to assess binaural TFS sensitivity. A temporal gap detection test (intertone burst delay) was also used to assess the sensitivity of temporal processes (Gallun *et al.*, 2014). Because gap discrimination can be performed either by using TFS information or envelope information carried by the auditory nerve (and refined by later processing), it is important to note that it is presently unclear which cue(s) are being evaluated or even whether or not gap discrimination evaluates the same cues among different listeners. Nevertheless, these three tests have been proposed previously as measures of TFS with potential clinical utility (Hoover *et al.*, 2019) and, thus, that category label is retained here for ease of reference.

The preferential tuning of auditory cortical neurons to modulation, both over time and across frequency, has resulted in an increased focus on the potential explanatory power of STM perception (Kowalski *et al.*, 1996; Theunissen *et al.*, 2000; Shamma, 2001; Schonwiesner and Zatorre, 2009). All natural sounds can be characterized as a pattern of STM (Theunissen *et al.*, 2000; Theunissen and Elie, 2014), and the relationship between sinusoidal STM and speech stimuli has been appreciated for some time (e.g., van Veen and Houtgast, 1985). This has led to a number of studies exploring sensitivity to spectral modulation, temporal modulation, and STM both for nonspeech stimuli (e.g., Whitefield and Evans, 1965) and speech stimuli (Bernstein *et al.*, 2013; Mehraei *et al.*, 2014; Venezia *et al.*, 2019) as central processes that exist beyond basic audibility (Gallun and Souza, 2008). Studies using STM in participants with suprathreshold hearing loss have found that an extra 40% of the variance of speech-in-noise performance can be accounted for by these evaluations beyond the 40% accounted for by the audiogram alone (Bernstein *et al.*, 2013; Mehraei *et al.*, 2014). Thus, this study included tests for temporal, spectral, and STM sensitivities, all of which are largely absent from the clinic.

Because the accurate identification of an acoustic target in competition is considered fundamental to auditory perception and scene analysis beyond peripheral audibility (Shinn-Cunningham, 2008; Moore *et al.*, 2014; Bronkhorst, 2015), tests were included that assess the capacity of the system to select relevant information and suppress test-irrelevant interference. The notched-noise method (Patterson, 1976; Moore and Glasberg, 1990) evaluates the detection of a tone presented in competition with noise either with or without a spectral notch around the target frequency. This test allows the evaluation not only of peripheral frequency selectivity but also frequency processing efficiency (Patterson, 1976; Moore and Glasberg, 1990; Stone *et al.*, 1992; Bergman *et al.*, 1992). To address auditory scene analysis, including speech and binaural processing, spatial release from masking (SRM; Marrone *et al.*, 2008; Gallun *et al.*, 2013; Jakien *et al.*, 2017; Jakien and Gallun, 2018) was assessed using the coordinate response measure (CRM) corpus (Bolia *et al.*, 2000). Following the methods of Gallun *et al.* (2013), speech understanding was assessed both with speech maskers colocated with the target speech in simulated space, as well as with the maskers separated from the target by 45 deg in simulated space. These tests independently assess speech understanding in competition under different stimulus conditions, and the difference between the scores on the two provides a measure of the ability of an individual listener to benefit from spatial differences between target and masking stimuli.

The purpose of this study was to determine the degree to which this preliminary PART battery is capable of reproducing standard laboratory results in a population of young, normal-hearing adults. To this end, the reliability of threshold estimation (test-retest) and the degree to which estimates obtained from PART approximate those reported in the literature for the same tests were both evaluated. Additionally, to address the robustness of results to different listening conditions, we evaluated the extent to which test measures were consistent across the use of different headphone types and under different ambient noise conditions. Ultimately, the goal of this work is to generate a normative dataset that could be used in a range of contexts from research to the clinic.

To accomplish these goals, data were collected from young normal-hearing students under similar conditions to previous validation work from our group (Gallun *et al.*, 2018) with repeated tests using circumaural headphones (repeatability condition), by means of both passive and active noise-attenuating headphones in a silent environment (headphone condition) and in the presence of recorded cafeteria noise (noise condition). First, the results addressing measurement reliability (test-retest) are presented. Second, the relation to the relevant literature is examined. Third, the effects of the experimental manipulations involving headphones and background noise are estimated. Overall, results show that PART produces repeatable threshold estimates consistent with those that have been reported previously in the laboratory across different listening conditions. These data serve as validation that accessible auditory hardware (consumer-grade tablet and headphones) can be used to test auditory function with sufficient precision to reproduce the thresholds obtained using laboratory-grade equipment. This dataset also provides a distribution of thresholds that can now be used as a normative baseline against which auditory dysfunction can be identified in future work.

## II. METHODS

### A. Participants

The listeners were 150 undergraduate students from the University of California, Riverside [42 male, *M* age = 19.6 yr, standard deviation (SD) = 2.31 yr] who received course credit for their participation. All participants reported normal hearing and vision and no history of psychiatric or neurological disorders. They provided signed informed consent as approved by the University of California, Riverside Human Research Review Board. In alignment with our goal to evaluate “normal” auditory processing, we rejected thresholds that deviated more than three SD from the mean of each assessment from the results presented in the main manuscript. A full dataset with the thresholds of all participants is included in a form suitable for further analysis, and analyses and plots with the full dataset are included in the supplementary materials (see Fig. S1 and Table ST1).^2^

**FIG. 1.**
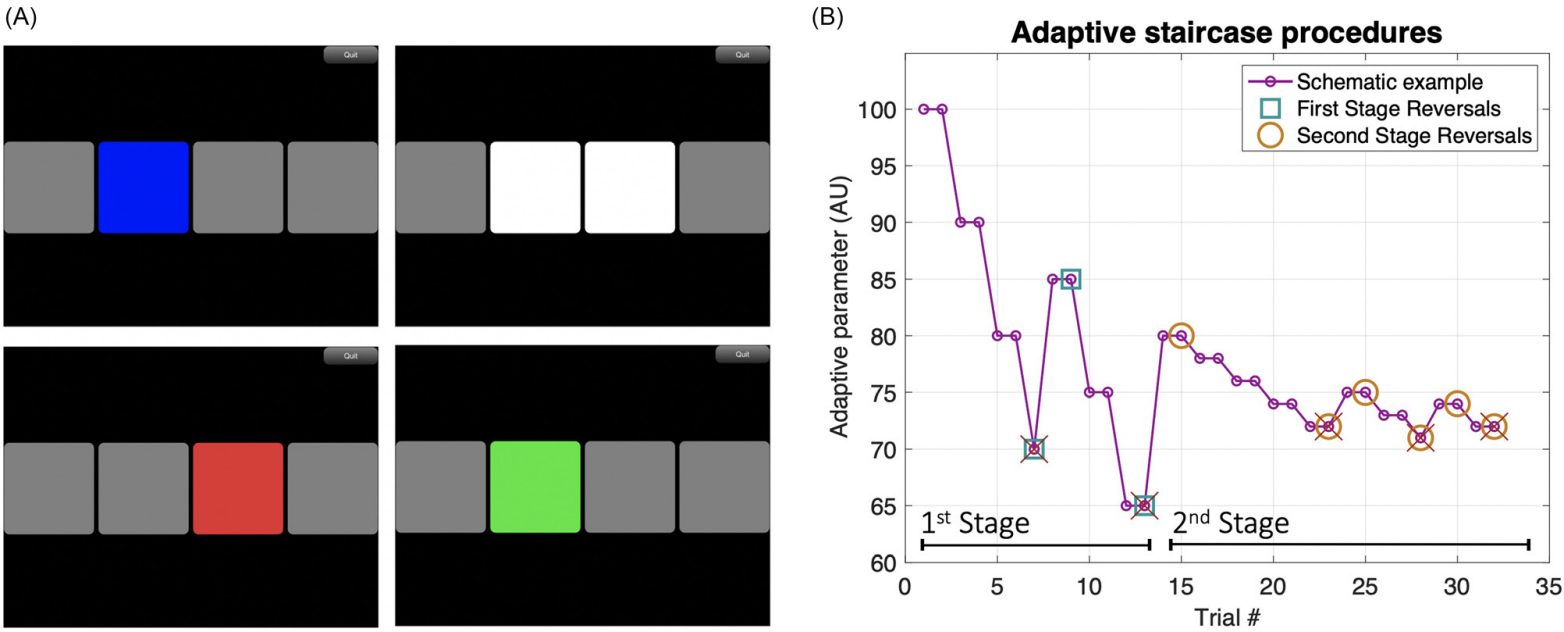
(Color online) In (A), each panel represents a screenshot taken from PART while on a two-cue, two-alternative forced-choice test. Each box is lit up sequentially in blue, emitting a sound (top-left). After all intervals were played, the two alternatives in the middle became available for response (top-right). Feedback is shown by the color code (red = wrong; bottom panels). In (B), we present a schematic example of the adaptive staircase procedures used. The difference in the magnitude of steps between staircase stages and the unequal step sizes going up/down can be easily observed in this example. Incorrect trials are marked with crosses and reversals are marked with either squares (first stage) or circles (second stage). Arbitrary units were selected as adaptive parameter values for descriptive purposes only.

### B. Materials

All procedures were conducted using standard iPad tablets (Apple Inc., Cupertino, CA) running the PART application with stimuli delivered via either Sennheiser 280 Pro headphones (Sennheiser electronic GmbH & Co. KG, Wedemark, Germany), which are rated to have a 32 dB passive noise attenuation with an 8 Hz–25 kHz frequency response, or Bose (active) noise cancelling Quiet Comfort 35 wireless headphones (Bose Corporation, Framingham, MA) set to the high noise cancelling setting. Output levels were calibrated for the Sennheiser headphones using an iBoundary microphone (MicW Audio, Beijing, China) connected to another iPad running the NIOSH Sound Level Meter application (SLM app)^3^ as described in Gallun *et al.*, 2018). The SLM app and iBoundary microphone system were calibrated with reference to measurements made with a Head and Torso Simulator with Artificial Ears (Brüel and Kjær Sound and Vibration Measurement A/S, Nærum, Denmark) in the anechoic chamber located at the Virginia Office of Rehabilitation Research and Development (VA RR and D) National Center for Rehabilitative Auditory Research (NCRAR). Similar testing of the Bose system revealed that the method used, which did not involve changing the calibration settings when the headphones were changed, resulted in an overall reduction in the mechanical output level at the ear of 14 dB but with no distortions in the time or frequency domain. The levels described here and used throughout the study refer to the calibrated Sennheiser system.

### C. Procedure

In each session, participants sat in a chair inside a double-walled sound-treated room and listened through a set of headphones connected to an iPad (Apple Inc., Cupertino, CA) running PART. Tests were self-administered with text-based instructions delivered within the PART application. Responses were collected via digital buttons presented on the iPad touch screen. Most tasks employed a two-cue two-alternative forced choice (two-cue two-AFC) procedure where four intervals are presented in an audio-visual sequence with inter-stimulus-intervals (ISI) of 250 ms [Fig. 1(A), top-left]. The first and last stimuli were standard cues, and participants made a choice between the two alternatives presented in the second and third intervals [Fig. 1(A), top-right]. Participants responded by touching the second or third square on the screen. The selected square then flashed either green (correct) or red (incorrect) as response feedback [Fig. 1(A), bottom] before proceeding to the next trial (1 s ITI). This two-cue two-AFC task, which is identical to the one used in Souza *et al.* (2020), has the advantage that unlike a two-interval or three-interval task, the target is always preceded and followed by a standard stimulus. This allows the task to be performed by comparing information either forward or backward in time. This is important as it is known that sensory comparisons are more difficult if they must be performed to a following standard rather than to a preceding standard especially for older listeners (Gallun *et al.*, 2012). A two-cue two-AFC design, thus, helps ensure that if in the future differences are found between the normative data reported here and data from other patient groups, differences will be less likely to reflect the influences of attention or memory and more likely to reflect actual differences in the ability to make sensory comparisons. The one task that differed in procedure was the SRM task, which uses a colored number grid to respond and has a fixed progression of difficulty (see the details below).

The tasks using the two-cue two-AFC procedure adjusted difficulty using a two-stage two-down one-up staircase procedure. The first stage used large steps for three reversals before moving on to the second stage, whcih used smaller steps (1/5 the size of the first stage) and terminated after six reversals. Further, to help ensure that after incorrect responses, participants were provided with easier exemplars, steps up were larger than steps down with a 2:1 step-size ratio in the repeatability condition, and 1.5:1 step-size ratio in the headphone and noise conditions. Thresholds were estimated from the geometric mean of the second-stage reversals. A general schematic of the adaptive staircase procedures is included in Fig. 1(B). This combination of up-down rule and step-size ratio results in a threshold estimate that asymptotically targets the stimulus level corresponding to 81.7% correct for 2:1 and 77.5% correct for 1.5:1, comparable to a 79.4% targeted by a typical three-down one-up staircase with equal steps up and down (Levitt, 1971; García-Pérez, 2001; see the supplementary materials for the comparison across procedures^2^). While unequal step sizes are common in audiometric testing (ANSI 3.21, 2004; ISO 8253–1, 2010), there are few who have followed the suggestion of García-Pérez (2011) in adopting the use of unequal step sizes when designing efficient staircase methods. The goal is to minimize the influence of task and listener factors that can result in thresholds deviating from the asymptotic target point (García-Pérez, 1998, 2001). Designing optimal methods for the clinical translation of laboratory procedures is a continued area of research by our group (e.g., Hoover *et al.*, 2019). The exploration of different ratios of unequal step sizes reported here represents an initial foray into this question.

Each session of the experiment began with a monitored screening test, which presented ten trials of a 2 kHz tonal target signal at 45 dB sound pressure level (SPL) in the environmental settings relative to each condition. In cases where participants failed to respond accurately on at least nine of the ten trials, instructions were repeated in isolation from other participants to ensure that the task was properly understood. All of those participants who needed to restart the testing reported that they did not realize that the tone would be presented at a fairly low level. Once properly prepared for the stimuli to be at 45 dB SPL, all participants were able to detect the 2 kHz tone with at least 90% accuracy. At this point, all participants moved on to complete two assessments involving the detection of the same 2 kHz tone but now presented in noise maskers with or without a spectral notch (described in detail below). Then, participants were pseudo-randomly assigned to complete the remaining eight assessments (details described below) in three blocks of testing organized by test type: TFS, (three assessments), STM (three assessments), and the second half of targets in competition (SRM; two assessments). All assessments were preceded by five nonadaptive practice trials at a high point in their respective staircase where target stimuli were easily detectable. Participants were encouraged to take small breaks between testing blocks. All three test blocks were given during each session. The ten assessments in the testing block took around 5 minutes each, resulting in test sessions of around 50 minutes. The second session was always conducted on a different day, which was no longer than a week after the first session. Test sessions involved up to three participants seated next to each other in a single room, listening and responding independently. In general, listeners received minimal instructions regarding the proper placement of the headphones and adherence to the brief written instructions automatically delivered by PART. The full verbal and written instructions given to each listener are provided in the supplementary materials.^2^

### D. Stimuli

**FIG. 2.**
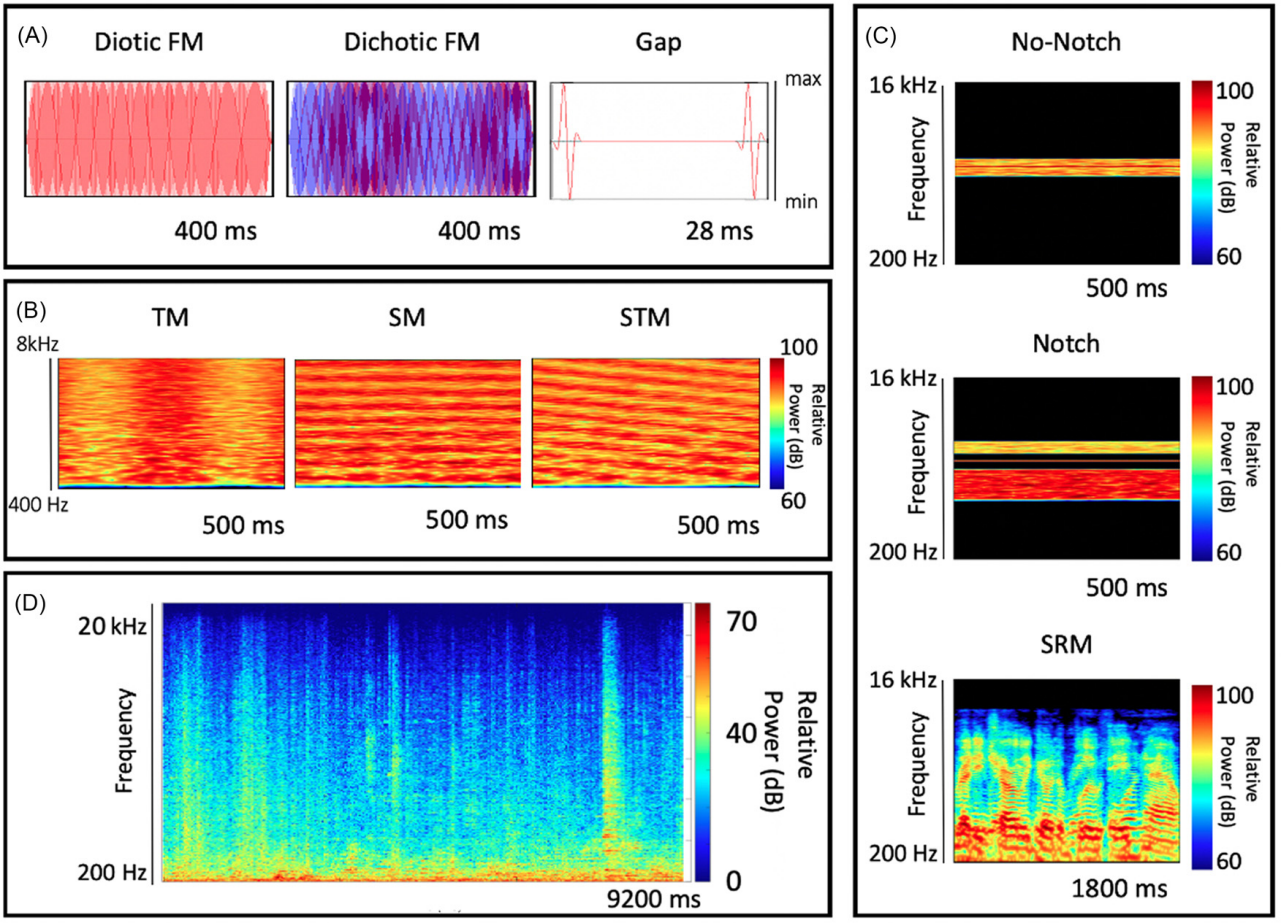
(Color online) Visual representations of the stimuli employed for each assessment grouped by sub-battery are shown in (A)–(C). Amplitude envelopes for the TFS sub-battery are shown in (A) and spectrograms for the rest of the assessments are shown in (B) and (C). A representative nine-second segment of the cafeteria noise utilized for the noise condition is shown in (D). The total recording had a duration of 11 min and was played in a continuous loop during testing.

Visual examples of the stimuli used in each assessment are shown in Fig. 2.

#### 1. TFS [Fig. 2(A)]

##### a. Temporal gap.

This gap discrimination task (Gallun *et al.*, 2014; Hoover *et al.*, 2019) compares a target signal that consisted of a diotically presented temporal gap placed between two 0.5 kHz tone bursts of 4 ms played at 80 dB SPL to standards that consisted of both tone bursts sequentially with no gap between them. The adaptive parameter was an intertone burst delay with an initial value of 20 ms. The staircase adapted on an exponential scale with first stage step-size (down) of 2^1/2^ and second stage step-size (down) of 2^1/10^ with a minimum of 0 ms and a maximum of 100 ms.

##### b. Diotic frequency modulation.

This FM detection task (Grose and Mamo, 2012; Whiteford and Oxenham, 2015; Whiteford *et al.*, 2017; Hoover *et al.*, 2019) compares a target diotic frequency modulation rate of 2 Hz to standards that consisted of a pure tone carrier frequency randomized between 460 and 550 Hz, each presented at 75 dB SPL for 400 ms. Randomization of the carrier frequency of standards ensures that the test cannot be successfully conducted by a simple pitch cue. The adaptive parameter was modulation depth with an initial value of 6 Hz. The staircase adapted on an exponential scale with first stage step-size (down) of 2^1/2^ and second stage step-size (down) of 2^1/10^ with a minimum of 0 and a maximum of 10 000 Hz.

##### c. Dichotic frequency modulation.

This frequency modulation (FM) detection task (Grose and Mamo, 2012; Hoover *et al.*, 2019) uses a stimulus first developed by Green *et al.* (1976), which creates a continuously shifting interaural phase difference (IPD) in the target interval. The task compares a target signal consisting of a frequency modulation rate of 2 Hz that is inverted or anti-phasic between the ears to standards that consisted of a pure tone carrier frequency randomized between 460 and 550 Hz, each presented at 75 dB SPL for 400 ms. The adaptive parameter was the modulation depth (which determines the size of the IPD) with an initial value of 3 Hz. The staircase adapted on an exponential scale with first stage step-size (down) of 2^1/2^ and second stage step-size (down) of 2^1/10^ with a minimum of 0 Hz and a maximum of 10 000 Hz.

#### 2. Spectro-temporal sensitivity [Fig. 2(B)]

All stimuli for these tasks involved a broadband noise that was either unmodulated (the standard) or modulated temporally, spectrally, or spectro-temporally, depending on the task (described below). The unmodulated standard consisted of flat-frequency broadband noise with a frequency range of 0.4–8 kHz. Stimuli were generated in the frequency domain using the maximum number of components allowed by a 44.1 kHz sampling rate with random amplitude and phase values, presented at 65 dB SPL for 500 ms. Modulation was applied on a logarithmic amplitude scale (dB), and the modulation depth was measured from the middle of the amplitude range to the peak amplitude as described in Isarangura *et al.* (2019). The stimuli were generated between trials using a custom algorithm developed for PART.

##### a. Temporal modulation (TM).

The TM detection task (Viemeister, 1979) compares a target with sinusoidal temporal AM at a rate of 4 Hz to the unmodulated standard. The adaptive parameter was modulation depth in dB. The staircase adapted linearly in dB with first stage step-size (down) of 0.5 dB and second stage step-size (down) of 0.1 dB with a minimum of 0.2 dB Hz and a maximum of 40 dB.

##### b. Spectral modulation (SM).

The SM detection task (Hoover *et al.*, 2018) compares a target with a sinusoidal SM with random phase at a rate of 2 cycles per octave (c/o) to an unmodulated standard. The adaptive parameter was THE modulation depth in dB, which was adaptively varied as in the TM task.

##### c. STM.

This STM detection task (Bernstein *et al.*, 2013; Mehraei *et al.*, 2014) uses stimuli similar to the TM AND SM tasks described above but compares a target with both 2 c/o SM and 4 Hz AM to standards that consisted of flat-frequency broadband noise. The resulting STM was randomly assigned to move upward or downward in frequency over time on each trial. The adaptive parameter was modulation depth in dB, which was varied as in the TM and SM tasks.

#### 3. Targets in competition [Fig. 2(C)]

##### a. No-Notch Condition.

This abbreviated notch-noise method is adapted from Moore (1987) and measures the ability of the listener to detect a target 2 kHz pure tone presented at 45 dB SPL in only one of the four intervals. The masking noise, which occurred on all intervals, consisted of 10 000 sinusoidal components distributed exponentially (-3 dB/octave) centered on the target frequency with a bandwidth of 1600 Hz (1.2–2.8 kHz) presented for 500 ms. The adaptive parameter was the root-mean-square (RMS) level of the noise, measured in dB. The staircase started with a noise level of 35 dB SPL and adapted on a linear scale with first stage step-size (down) of 6 dB SPL and second stage step-size (down) of 2 dB SPL with a minimum of 25 dB SPL and a maximum of 90 dB SPL.

##### b. Notch condition.

This condition was identical to the no-notch condition with the exception that a spectral notch of 0.8 kHz was introduced, increasing the bandwidth of the masker such that it covered two frequency ranges: 0.8–1.6 kHz and 2.4–3.2 kHz, leaving a 0.8 kHz notch centered on 2 kHz, which was the frequency of the target to be detected. The adaptive parameter was the RMS masker level, which again had a starting value of 35 dB SPL. The staircase adapted in the same manner as in the no-notch condition. This condition is equivalent to a notch width of 0.2 times the center frequency of 2 kHz measured from center to the nearest edge of the noise as described by Moore (1987). The difference in threshold with the no-notch condition can be taken as an index of frequency (spectral) resolution.

##### c. SRM colocated.

The three-talker speech-on-speech masking method of Marrone *et al.* (2008) as adapted for progressive tracking by Gallun *et al.* (2013) was used to measure the ability of listeners to identify keywords of a target sentence in the presence of two masking sentences. Using a color/number grid (four colors by eight numbers) participants identified two keywords (a color and a number) by selecting the position indicated by the keywords spoken by the target talker, which was a single male talker from the CRM corpus (Bolia *et al.*, 2000), presented from directly in front of the listener in a virtual spatial array. Target sentences all included the call sign “Charlie” and two keywords: a number and a color. Targets were fixed at a RMS level of 65 dB SPL. The target was presented simultaneously with two maskers, which were male talkers uttering sentences with different call signs, colors, and numbers in unison with each other and the target. All three sentences were presented from directly in front of the listener (colocated). Progressive tracking included 20 trials in which the maskers progressed in level from 55 to 73 dB SPL in steps of 2 dB every two trials as reported in Gallun *et al.* (2013), resulting in two responses at each of ten target-to-masker ratios (TMRs). The threshold TMR was calculated following Gallun *et al.* (2013) by subtracting the number of correct responses from 10 dB, resulting in values between 10 dB for no correct responses to −10 dB for all correct responses. Negative TMR thresholds indicate that threshold performance (roughly 50% correct) could be achieved when the target was at a lower level than the maskers, whereas positive thresholds indicate that the maskers needed to be lower in level than the target.

##### d. SRM separated.

The stimuli were identical to those in the colocated condition with the exception that the maskers were presented from 45 deg to the left and right of the target talker. Responses were again given in the context of a color/number grid (four colors by eight numbers) and participants had to select the position indicated by the target signal. The masker level again progressed every other trial from 55 to 73 dB SPL in steps of 2 dB as reported in Gallun *et al.* (2013), and the threshold TMR was again estimated by subtracting the number correct from 10 dB. The spatial release metric was estimated by subtracting the threshold in the separated test from the threshold in the colocated test, resulting in values between −20 and 20 dB with 0 dB indicating no SRM, positive values indicating improvements in performance with spatial separation, and negative values indicating reduced performance with spatial separation.

### E. Experimental design

The study consisted of three different conditions targeted to evaluate the repeatability of PART procedures in a variety of settings. These conditions were run sequentially on three different groups of participants.

(1) *Repeatability condition—*The first 51 students enrolled were tested with Sennheiser 280 Pro headphones (Sennheiser electronic GmbH and Co. KG, Wedemark, Germany) for both sessions and used 2:1 up/down step-size ratio in the staircase.
(2) *Headphone condition (in silence)*—The next 51 participants enrolled were tested with different headphones (Sennheiser 280 Pro vs Bose Quiet Comfort 35; Bose Corporation, Framingham, MA) with the order counterbalanced between participants and used a 1.5:1 up/down step-size ratio.
(3) *Noise condition*—The next 48 participants enrolled were tested using the same procedure as in the headphone condition but with recorded cafeteria noise played at 70 dB SPL. The noise was recorded in a local coffee shop, edited to remove silent gaps between recordings and transient recording noise at the beginning and ends of the recordings, and then bandpass filtered between 20 and 20 000 Hz. The coffee shop noise contained a large number of sound sources at all times, including both speech and environmental sounds. A spectrogram of a representative segment is shown in Fig. 2(D). Sound files, after processing, were 11 min in duration and were played on a loop through two loudspeakers placed 30 cm apart from each other and positioned in the center of the back of the test room, between 5 and 6 m behind the three listeners.

## III. RESULTS

Results are divided into sections for the purpose of clarity. First, the results for each test, session, and condition are presented (Sec. III A). Then, issues of test-retest reliability are addressed (Sec. III B), and the consistency of results for each test in comparison to previously reported measures are described (Sec. III C). Finally, the effects of headphones and noise are addressed (Sec. III D). The full dataset is provided in the supplementary materials for transparency and to encourage replotting,^2^ comparison with future and past data, and/or reanalysis.

### A. Overview

**FIG. 3.**
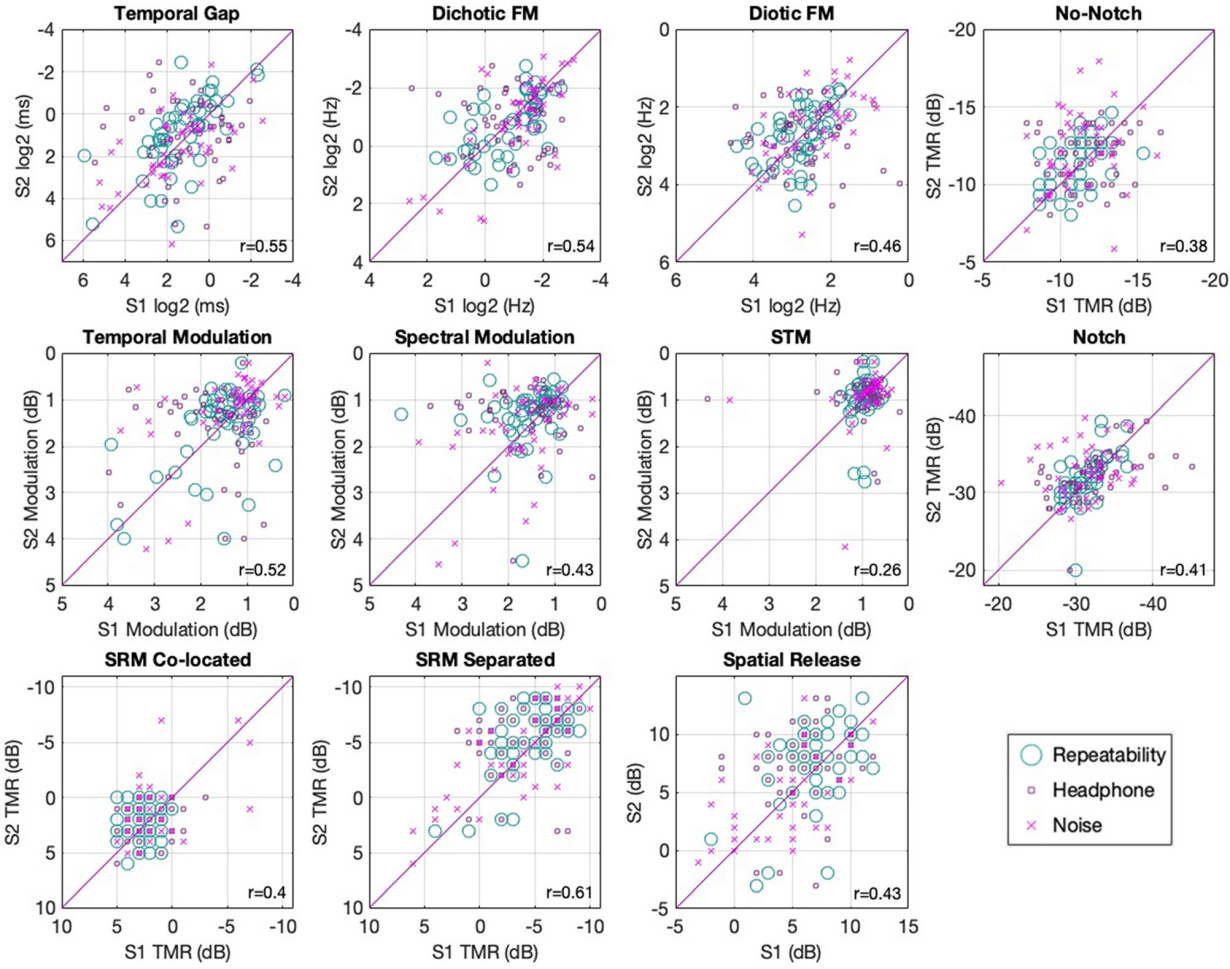
(Color online) Scatter plots of session 1 vs session 2 for the ten assessments. All axes are oriented to show better performance values away from the origin. Correlations are indicated in the lower right of each panel. Different markers illustrate the different conditions.

An overview of the results can be seen in Fig. 3, which plots data for each test for each participant in each condition and session. Figure 3 shows the relationship between estimated thresholds in sessions 1 and 2 and substantial overlap of performance between conditions. This interpretation is consistent with summary statistics for each test shown as a function of condition and session in Table I. Due to the high consistency between conditions, the “main effects” were first analyzed by collapsing the data across condition (Secs. III B and III C) before addressing the effects of condition (Sec. III D). By combining data across conditions, a large normative dataset could be constructed, consisting of ∼150 participants per test. Consistent with this goal of showing a normative sample, outlier rejection was performed by removing all data that exceeded three SDs from the mean for any condition of any task. The implications of this decision are addressed in Sec. IV (discussion). The supplementary materials are provided and demonstrate the robustness of the results to different choices of outlier rejection, as well as replotting data from Fig. 3 with outliers included and labeled.^2^

**TABLE I.** Mean thresholds and SDs for the ten assessments utilized plus the derived spatial release metric across all three conditions and their aggregate. Data are presented in PART's native measurement units except for the targets in competition tests, which have been converted to TMR. The first row of each test shows session 1 and session 2 (S2).

Test	Repeatability *M* (SD)	Headphone *M* (SD)	Noise *M* (SD)	All Cond. *M* (SD)	Units
Gap	2.4 (2.9)	2.17 (3.2)	2.83 (3.41)	2.46 (3.15)	Gap length (ms)
S2	1.8 (3.34)	2.08 (3.04)	3.008 (3.07)	2.26 (3.18)	
Dichotic FM	0.55 (2.09)	0.54 (2.18)	0.45 (2.44)	0.51 (2.23)	Modulation depth (Hz)
S2	0.57 (2)	0.47 (2.37)	0.52 (2.77)	0.52 (2.37)	
Diotic FM	7.07 (1.57)	6.55 (1.88)	5.48 (1.78)	6.35 (1.75)	Modulation depth (Hz)
S2	6.71 (1.65)	5.65 (1.76)	5.82 (1.89)	6.05 (1.77)	
TM	1.58 (0.77)	1.64 (0.82)	1.42 (0.85)	1.55 (0.81)	Modulation depth (dB)
S2	1.58 (0.85)	1.46 (0.85)	1.21 (0.84)	1.42 (0.85)	
SM	1.57 (0.61)	1.55 (0.75)	1.71 (0.82)	1.61 (0.72)	Modulation depth (dB)
S2	1.33 (0.63)	1.37 (0.75)	1.64 (0.92)	1.44 (0.78)	
STM	0.94 (0.17)	0.97 (0.59)	0.93 (0.53)	0.95 (0.46)	Modulation depth (dB)
S2	0.97 (0.49)	0.98 (0.55)	0.94 (0.62)	0.96 (0.55)	
No-notch	−11.28 (1.38)	−11.82 (1.92)	−11.15 (2.14)	−11.43 (1.84)	TMR (dB)
S2	−11.74 (1.71)	−12.88 (2.39)	−12.16 (2.48)	−12.26 (2.24)	
Notch	−31.4 (2.27)	−32.26 (4.52)	−31.29 (3.82)	−31.67 (3.64)	TMR (dB)
S2	−31.91 (3.02)	−32.71 (3.61)	−32.77 (3.31)	−32.44 (3.32)	
SR colocated	2.33 (1.36)	2.07 (1.67)	1.92 (2.77)	2.12 (1.96)	TMR (dB)
S2	2.01 (1.51)	1.84 (1.96)	1.34 (2.74)	1.76 (2.08)	
SR separated	−4.58 (2.64)	−3.58 (2.93)	−3.57 (4.23)	−3.91 (3.32)	TMR (dB)
S2	−5.36 (2.94)	−5.5 (2.64)	−4.19 (3.86)	−5.04 (3.2)	
Spatial release	6.94 (2.78)	5.66 (2.9)	4.57 (3.72)	5.8 (3.24)	Separated - colocated (dB)
S2	7.34 (3.4)	7.35 (2.71)	4.67 (3.41)	6.58 (3.37)	

### B. Test-retest reliability

Test-retest reliability for the two sessions performed for each assessment in each experimental condition was evaluated using three metrics: limits of agreement (LoA; Altman and Bland, 1983; Bland and Altman, 1999), correlation, and *t*-tests. Each of these measures provides a different but complementary perspective on test reliability. The LoA analysis is considered a gold standard analysis as it provides information regarding both agreement and bias (e.g., systematic difference between sessions). Correlations are included to provide a measure of within-subject consistency that ignores systematic effects of session, which can be important for research studies that seek to correct for effects of session. Last, *t*-tests were calculated as a function of session to help determine the reliability of effects of session.

#### 1. Test-retest reliability using LoA

**FIG. 4.**
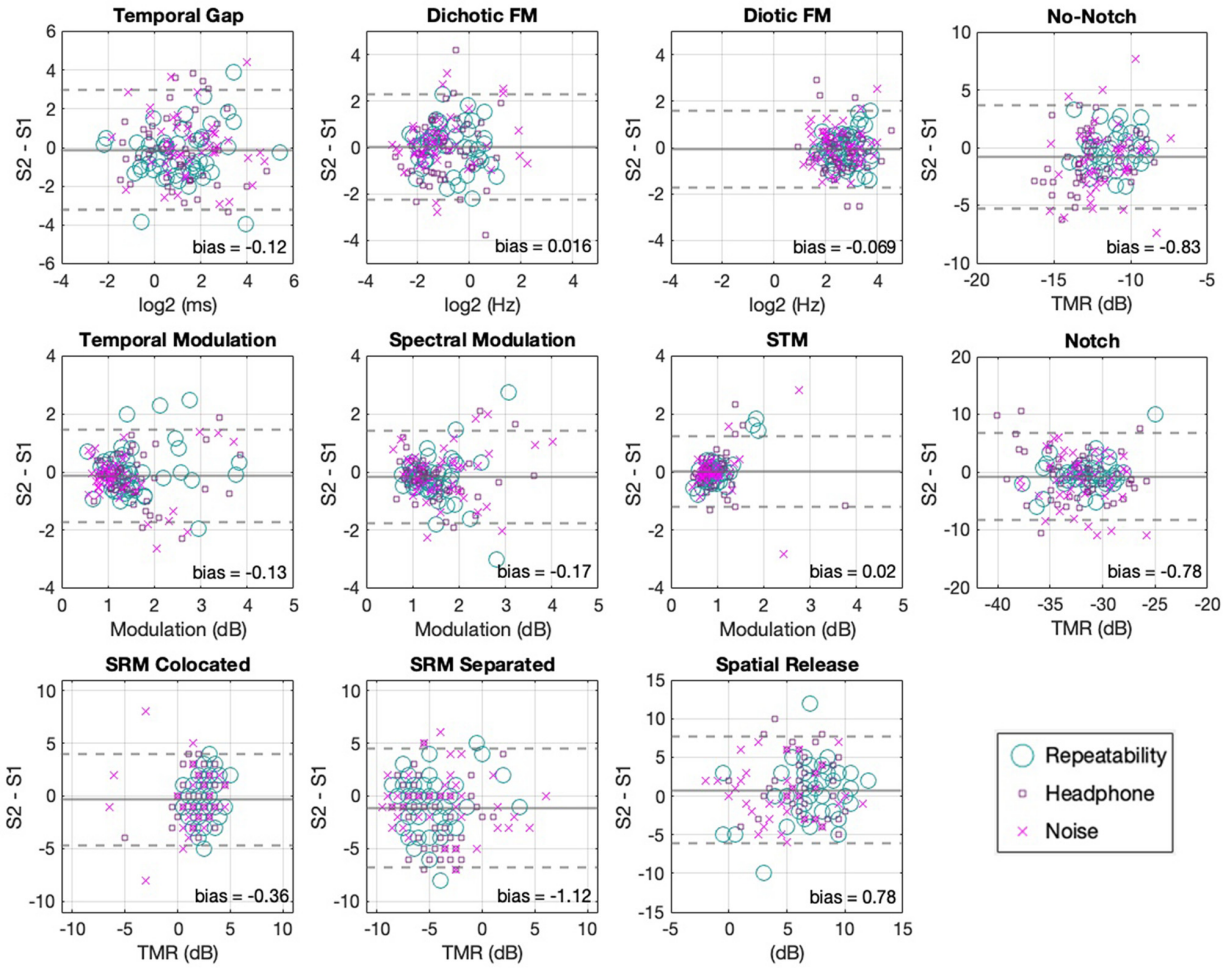
(Color online) The mean threshold of both sessions is plotted against their difference, showing the LoA between sessions for all tests. The solid lines indicate the mean difference between sessions. Dotted lines indicate the 95% LoA. Solid lines that fall below zero indicate better performance on session 2 (except the spatial release metric). Different markers illustrate the different conditions.

For these data, LoA were considered to be a more informative measure of reliability than the normalized correlation as between-subject variability for a sample that consisted solely of young listeners without hearing problems was anticipated to be small. Correlations, although more common in the auditory literature, are known to depend heavily on between-subject variability and measurement range (Altman and Bland, 1983; Bland and Altman, 1999). LoA plots for the estimated thresholds for each assessment are shown in Fig. 4 and the statistics are shown in Table II. This analysis is based on the evaluation of performance across sessions (mean of test and retest) as a function of their differences. LoA plots can be used to determine the extent to which learning effects are present, which would represent shifts toward better performance across sessions, the region where 95% of the difference between test and retest is expected to lie, and whether these statistics hold for different levels of performance (homoscedasticity).

**TABLE II.** LoA and within-subject significance testing for the ten assessments utilized at two time points. Negative values on the bias column indicate better performance on the second session except on the spatial release metric, which is the only scale in which larger magnitudes indicate better performance. “*” indicates significance at α = 0.05.

Test	Bias	LoA	Units	*r* (*p*)	*t* (*p*)	Cohen's *d*	df
Gap	−0.12	[−3.2 to 2.96]	log 2 (ms)	0.55 (<0.01)*	0.94 (0.34)	−0.07	145
Dichotic FM	0.01	[−2.2 to 2.2]	log 2 (Hz)	0.53 (<0.01)*	−0.17 (0.86)	0.01	147
Diotic FM	−0.06	[−1.73 to 1.59]	log 2 (Hz)	0.46 (<0.01)*	0.97 (0.33)	−0.08	144
TM	−0.13	[−1.73 to 1.47]	*M* (dB)	0.52 (<0.01)*	1.94 (0.054)	−0.15	145
SM	−0.16	[−1.75 to 1.41]	*M* (dB)	0.58 (<0.01)*	2.46 (0.01)*	−0.22	140
STM	0.01	[−1.2 to 1.2]	*M* (dB)	0.26 (<0.01)*	−0.29 (0.76)	0.03	136
No-notch	−0.83	[−5.3 to 3.6]	TMR (dB)	0.38 (<0.01)*	4.37 (<0.01)*	−0.4	144
Notch	−0.77	[−8.2 to 6.6]	TMR (dB)	0.40 (<0.01)*	2.44 (0.01)*	−0.22	142
Colocated	−0.36	[−4.7 to 3.9]	TMR (dB)	0.39 (<0.01)*	1.95 (0.052)	−0.17	142
Separated	−1.12	[−6.7 to 4.5]	TMR (dB)	0.61 (<0.01)*	4.47 (<0.01)*	−0.34	147
Spatial release	0.78	[−6.1 to 7.6]	dB	0.47 (<0.01)*	−2.62 (<0.01)*	0.23	140

In order to facilitate visual inspection and comparisons across different tests, TFS tests were transformed from PART's output units (either Hz or ms) to log_2_ units and target in competition tests were converted to TMRs (subtracting the target level from PART's masker level outputs). The mean across sessions is plotted on the *x* axis to give a single point estimate for each participant in terms of their estimated threshold, thus, showing between-subject variability of threshold estimation. The difference between sessions is plotted on the *y* axis to give a single point estimate of the magnitude of deviation between sessions, thus, showing within-subject variability of the estimated threshold. The mean of these differences is plotted as a straight line across the *x* axis, and its distance from zero (zero = perfect agreement) represents the main point estimate of the measurement's systematic bias across sessions. The 95% LoA [±1.96 SD (difference between sessions)] are plotted as dotted lines and indicate an estimate of the region in which we may expect to observe 95% of the within-subject, between-session differences of threshold estimation.

As can be observed in Fig. 4 and Table II, the mean difference between sessions was close to zero in all of the tests, indicating little systematic bias. The 95% LoA for the frequency modulation tests were at modulation rates of approximately ±2 log_2_ (Hz; or between 0.2 and 4.8 Hz). For the gap detection task, the LoA were approximately ±3 log_2_ (ms; or between 0.1 and 7.7 ms). The LoA for the modulation detection tasks were approximately ±3 dB. For the speech tasks, the LoA are TMRs of approximately ±8 dB for the targets in competition tests. Precise values are reported in Table II. As can be seen in Fig. 4, the distribution of the threshold estimates had no salient asymmetries, and session differences were similar across different levels of performance (symmetry along the abscissa). It is worth noting, however, that more spread can be identified at worse performance levels for some individuals in some tests. This applies both to the full set of data points in each plot and also to the subset of each showing the different conditions. There was little systematic bias between sessions (symmetry along the ordinate), suggesting a similar measurement error for both sessions and that the poorer performance cases were expressed without a clear bias toward either session. This analysis demonstrates the range of alignment to be expected between different threshold estimates within subjects and indicates that PART produces minimally biased estimates at the group level (see Table II for relevant statistics).

#### 2. Correlations between sessions

Table II shows statistics, including the strength of association (Pearson *r*) between sessions. Significant correlations were observed for all the assessments. Overall, the relatively low correlation magnitudes reflect the warning of Altman and Bland (1983) that correlations are less informative to quantify reliability than LoA plots when performance is distributed across relatively narrow ranges of threshold estimates as was to be expected for young listeners without hearing problems. This is particularly clear in the case of the STM assessment (in the same scale as the SM and TM assessments) where the range of threshold values obtained was quite restricted. In this context, the reduced between-subject variability in relation to a particular within-subject variance will have an impact on *r* values, decreasing their magnitude.

#### 3. Repeated measures t-tests

To supplement the LoA plots as a test for whether learning or other factors gave rise to systematic changes in performance, thresholds were compared between sessions across all three conditions using repeated measures *t*-tests (see Table II). While there were statistically significant differences between sessions in the SM detection test and the tone-in-noise tests, these changes were quite small with magnitudes of less than 1 dB. The speech intelligibility test in the separated condition showed a significant difference of greater than 1 dB, which is consistent with the 1.58 dB difference previously reported by Jakien *et al.* (2017).

### C. Comparison with previously published results

While the above analyses demonstrate reliability of these PART assessments, it is possible that the rapid methods, the presence of noise, or consumer-grade equipment would result in deviations from the results expected based on the published literature. This section, therefore, compares the thresholds reported in Table I for all conditions averaged across sessions to those previously reported in the literature. This “grand mean” threshold estimate includes thresholds from all of our 150 participants minus rejected outliers and is included in Table III. As outlier rejection was conducted by removing any points that fell more than three SDs above or below the mean, the number of outliers rejected and from which conditions is also reported in this section (also in supplementary Table ST1^2^) so that this can be considered in the comparisons. Overall, threshold estimates align with previous reports within 1.6 SD (see Table III), and the number of outliers rejected was roughly consistent with the statistical expectation.

**TABLE III.** Summary of the similarities of the grand average thresholds estimated in the present study using PART and matched psychophysical tests from previous research. Plus or minus signs indicate values that are better or worse than previous reports, respectively. The number of signs indicates increases in terms of SDs, one sign indicates <1 SD and two signs indicate between 1 and 2 SD. Cases with both a plus and a minus sign indicate that different conditions or experiments reported previously are <1 SD above and below the threshold estimates in this study.

Assessment	Grand average *M* (SD)	Closest laboratory test	Distance in SD
Gap	2.36 (3.16) ms	Gallun *et al.*, 2014	-
Dichotic FM	0.52 (2.3) Hz	Grose and Mamo, 2012; Hoover *et al.*, 2019	-
Diotic FM	6.2 (1.76) Hz	Grose and Mamo, 2012; Hoover *et al.*, 2019	- -
TM	1.49 (0.83) *M* (dB)	Viemeister, 1979	-
SM	1.52 (0.75) *M* (dB)	Hoover *et al.*, 2015	++
STM	0.95 (0.51) *M* (dB)	Gallun *et al.*, 2018	±
No-notch	−11.85 (2.09) TMR (dB)	Patterson, 1976	- -
Notch	−32.06 (3.5) TMR (dB)	Patterson, 1976	- -
SR colocated	1.94 (2.03) TMR (dB)	Jakien *et al.*, 2017; Gallun *et al.*, 2018	±
SR separated	−4.47 (3.31) TMR (dB)	Jakien *et al.*, 2017; Gallun *et al.*, 2018	±
Spatial release	6.19 (3.32) (dB)	Jakien *et al.*, 2017; Gallun *et al.*, 2018	±

#### 1. TFS

Sensitivity to temporal processing was assessed with three different tests, temporal gap detection, dichotic FM, and diotic FM. For temporal gap detection, four cases were rejected as outliers (headphone condition 2, noise condition 2), leaving threshold values that closely resemble those found in the literature (*M* = 2.36 ms, SD *=* 3.16). For example, Schneider *et al.* (1994) reported thresholds of 3.8 ms (right ear) and 3.5 ms (left ear) on average, using 2 kHz tone bursts similar to the ones we used, however, their stimuli were delivered monaurally. Moreover, Hoover *et al.* (2019) reported thresholds of 1.45 ms using 0.75 kHz tone bursts. Gallun *et al.* (2014) used the most similar stimuli (tone bursts of 2 kHz) and obtained thresholds of 1.2 ms on average. All three of these studies used monaural presentation of their stimuli. Despite the differences in stimulus frequency and presentation style, all of these estimates lie within half of a SD from the PART dataset. The fact that the published data report smaller thresholds and the second run appeared to produce smaller thresholds in this study suggests that the differences with the published literature might be removed by providing additional practice in the form of multiple measurements as opposed to the single track on each test session used here.

For the frequency modulated tests (dichotic and diotic FM), thresholds were higher than those previously reported in the literature. For the dichotic FM test, two cases were rejected as outliers (repeatability condition 2). Thresholds in Hz (*M =* 0.52, SD *=* 2.29) are around 1 SD higher (on a logarithmic scale) than the 0.2 Hz found by Grose and Mamo (2012), the 0.15 Hz reported by Whiteford and Oxenham (2015), and the 0.19 Hz reported by Hoover *et al.* (2019).

For diotic FM, five cases were rejected as outliers (repeatability condition 1, headphone condition 3, noise condition 1). Thresholds in Hz (*M =* 6.19, SD *=* 1.76) were about 2 SD higher than reports by Grose and Mamo (2012) of 1.9 Hz, Whiteford and Oxenham (2015) of 0.75 Hz, Moore and Sek (1996) of 1.12 Hz, and Hoover *et al.* (2019) of 1.85 Hz, after conversion to Hz using the method of Witton *et al.* (2000) where appropriate. These differences in both FM tests are likely due to the difference in stimulus durations employed, which in the literature vary between 1000 ms (Moore and Sek, 1996) and 2000 ms (Whiteford and Oxenham, 2015). Here, the duration was set to 400 ms. This choice was based on the results of Palandrani *et al.* (2019), who showed that FM detection thresholds improve with the square-root of the stimulus duration. This predicts diotic FM thresholds of 3.6 Hz for the listeners here if durations that were comparable to those of Grose and Mamo (2012) had been used. Even after this correction, however, the thresholds obtained were about 1 SD worse, on average (on a logarithmic scale), after conversion to Hz using the method of Witton *et al.* (2000) where appropriate. As with the temporal gap assessment, it would not be surprising if repeated testing resulted in reduced thresholds, more similar to those reported in the literature.

#### 2. STM

Sensitivity to STM was assessed with three different tests, STM, SM, and TM. It is difficult to make exact comparisons with previously reported results in the literature without making a variety of transformations and ignoring several differences in methodology. The most important issue is the measurement of the modulation depth. Measurement depends on the scale (log or linear), the reference points (peak-to-valley or peak-to-midpoint), and the order in which the modulation operations are performed, among other factors (see Isarangura *et al.*, 2019). In this case, PART generated stimuli that were modulated on a logarithmic amplitude scale (dB) with modulation depth measured from the middle of the amplitude range to the peak amplitude. This differs from the method used by others, such as Hoover *et al.* (2018), who measured applied modulation that was sinusoidal on a dB scale but measured the amplitude as the difference from the maximum to the minimum rather than the midpoint. Still more different was Bernstein *et al.* (2013), who applied sinusoidal modulation on a linear scale and also measured the modulation depth from the maximum to the minimum. When the amplitude scale is linear, the threshold is expressed by transforming the modulation depth (m), which varies between 0 and 1, into dB units using the value 20 times log(m), which means that a fully modulated signal has a value of 0 dB and a modulation depth of 0.01 has a value of −40. These differ from the values used to express threshold using a log amplitude scale and, thus, Eq. (1) was used to convert the thresholds obtained with PART to 20 log(m) dB units as detailed in Isarangura *et al.* (2019),

$$20\;\;{\text{Log}}_{10}\left(\frac{10\left(\frac{m}{10}\right)-1}{10\left(\frac{m}{10}\right)+1}\right).$$ (1)

For STM at 4 Hz and 2 c/o, 13 cases were rejected as outliers (repeatability condition 2, headphone condition 3, noise condition 8). STM thresholds obtained [*M =* 0.95 (*M*) dB, SD *=* 0.51] were converted using Eq. (1) to [*M =* −19.28 20 log(*m*) dB, SD *=* 4.67] and closely resembled those previously reported in the literature. They were within a SD from those reported by Gallun *et al.* (2018) for five different testing sites (range −21.74 to −18.42 dB) and Chi *et al.* (1999; −22 dB). The obtained thresholds for STM are about 2 SD better than those reported by Bernstein *et al.* (2013; −14 dB). It is unclear why outlier rejection was higher in this task than in others, but this may indicate that this is an ability for which some listeners perform particularly poorly. It is worth noting that the supplementary materials, where these statistics are reported without outlier rejection, still show better performance than in the literature.^2^

For SM at 2 c/o, 9 cases were rejected as outliers (repeatability condition 1; headphone condition 4, noise condition 4). SM modulation depth thresholds (*M =* 1.52 dB, SD *=* 0.75) were converted using Eq. (1) to *M =* −15.34 20 log(*m*) dB, SD *=* 4.23 were better by about one SD than those reported by Hoover *et al.* (2018; −11.08 dB) and those reported by Davies-Venn *et al.* (2015; about −11 dB). These differences might be due to differences in modulation depth generation patterns or modulation depth metrics employed (see Isarangura *et al.*, 2019). Further, stimulus parameters like those of the noise carrier bandwidth or presentation level and test parameters, such as tracking procedure, varied across studies and so might account for the slight differences found. One reason to suspect that these methodological differences influenced performance is the fact that our listeners often outperformed the more practiced listeners in the other studies. Again, a higher number of outliers were observed but as can be seen in the supplementary materials, this did not account for the better performance in this study.^2^

For TM at 4 Hz, four cases were rejected as outliers (repeatability condition 1, noise condition 3). TM thresholds [*M =* 1.49 (M) dB, SD *=* 0.83], converted using Eq. (1) to *M =* −15.99 20 log(*m*) dB, SD *=* 4.34, were within half a SD of those reported by Viemeister (1979), which were −18.5 dB for four observers.

#### 3. Target identification in competition

*Tone detection in noise with and without a spectral Notch*—These tests evaluated the ability to detect a 2 kHz pure tone in competition with broadband noise either overlaying the target signal (no-notch condition) or with an 800 Hz spectral notch or protective region without noise (notch condition). We rejected five cases as outliers (headphone condition 1, noise condition 4) from the no-notch test and obtained thresholds of *M =* −11.85, SD *=* 2. In the case of the notch test, we rejected seven cases (headphone condition 1, noise condition 6) and obtained thresholds of *M =* −32.06, SD *=* 3.5. The notched-noise procedure has been widely used for the analysis of frequency selectivity in the cochlea (see Moore, 2012). Because of this, the emphasis of the literature has been on calculating detailed information about the shape of the auditory filter, and specific thresholds associated with each condition are typically not reported. In one of the few examples where thresholds are described directly, Patterson (1976) reported an average distance between the equivalent of our no-notch and notch conditions of about 24 dB for four participants. This is comparable to the mean distance we obtained here of 20.2 dB (SD = 2.9), and some of our participants did indeed produce thresholds similar to those of the four well-practiced listeners described in Patterson (1976). That some of our listeners were able to obtain the same thresholds as the well-trained listeners described in Patterson (1976) suggests that this may be a task for which training plays a fairly small role. This conclusion is supported by the finding, shown in Table II, that the thresholds in the first run were on average less than 1 dB higher than the thresholds obtained on the second run.

*Speech-on-speech competition*—These tests evaluated the discrimination of speech in the face of speech competition using variants of the SRM test described by Gallun *et al.* (2013). Two conditions were used, one where the speech-based competition was colocated in virtual space with the target speech (colocated) and one where the speech-based competition was located ±45 deg away from the target (separated) in simulated space. All values are reported in TMR dB units. In the case of the colocated condition, seven cases were rejected as outliers from the noise condition. Interestingly, these cases are mainly due to performance that was better than average by more than 3 SD (see supplementary materials, Fig. S1),^2^ which has been observed previously for the occasional younger listener with normal hearing. Colocated thresholds (*M* = 1.94 dB, SD = 2.03) closely resemble those reported by Gallun *et al.* (2018) across two testing sites (1.85 and 1.96 dB). Performance was slightly worse than predicted by the normative functions of Jakien and Gallun (2018), which are based on linear regression to the data from a variety of listeners varying in age and hearing loss. For a 20 year old with a pure-tone average (PTA) of 5 dB hearing level (HL), which seems appropriate for this sample, colocated thresholds averaged across two runs are predicted to be 1.2 dB, which is within 1 SD of what is observed.

In the separated condition, two cases were rejected as outliers (repeatability condition 1, noise condition 1). Separated thresholds (*M* = −4.47 dB, SD = 3.31) closely resembled those reported by Gallun *et al.* (2018) across two testing sites (−4.33 and −4.62 dB), all of which were higher on average than the predictions of the equation of Jakien and Gallun (2018), which predicts a threshold of −6.7 dB, which is still within 1 SD of those observed.

The difference between the separated and colocated conditions, a metric indicating the SRM effects, showed spatial release values (*M* = 6.19 dB, SD = 3.32) that again closely resembled the ones reported by Gallun *et al.* (2018) across two testing sites (6.19 and 6.57 dB) and were within 1 SD of the predictions of the regression equation of Jakien and Gallun (2018), which predicts 8.3 dB. Of note, the SRM magnitudes reported here are smaller than those reported for other similar tests already used in the clinic like the LiSN-S (Cameron and Dillon, 2007), which do not use synchronized concurrent masking and, thus, allow for better ear glimpsing (Brungart and Iyer, 2012) to contribute to the effect of release from masking (Glyde *et al.*, 2013).

### D. The effects of headphones and noise

To address the effects of headphone types with and without noise-attenuation technology and external noise conditions, main effects were evaluated by collapsing across tasks. To do so, composite scores were computed by normalizing each individual assessment relative to its mean and SD (a *z*-score transform), calculating a *z*-score for each listener in each assessment, and then averaging these normalized values across the ten assessments for each participant. *z*-scores and composite scores are included in the dataset that is available as part of the supplementary materials.^2^

LoA plots, Pearson correlations, and *t*-tests were then computed for the composite scores. Results are reported for each condition separately, and divided by headphone type. To test differences across experimental manipulations, a mixed-model analysis of variance (ANOVA) was used to compare composite scores across the factors of interest. The data for each condition as a function of test type are available in the supplementary materials.^2^

The internal reliability of the composite score was assessed by calculating Cronbach's α, which gave a score of 0.75. This indicates that the composite has strong internal reliability and, therefore, it can be appropriate to use it as a summary score. Of note, the composite score is not an attempt to reduce central auditory processing to a single construct. Rather, this measure is intended to address the effects of these experimental manipulations in an efficient manner across all the assessments in the battery.

**FIG. 5.**
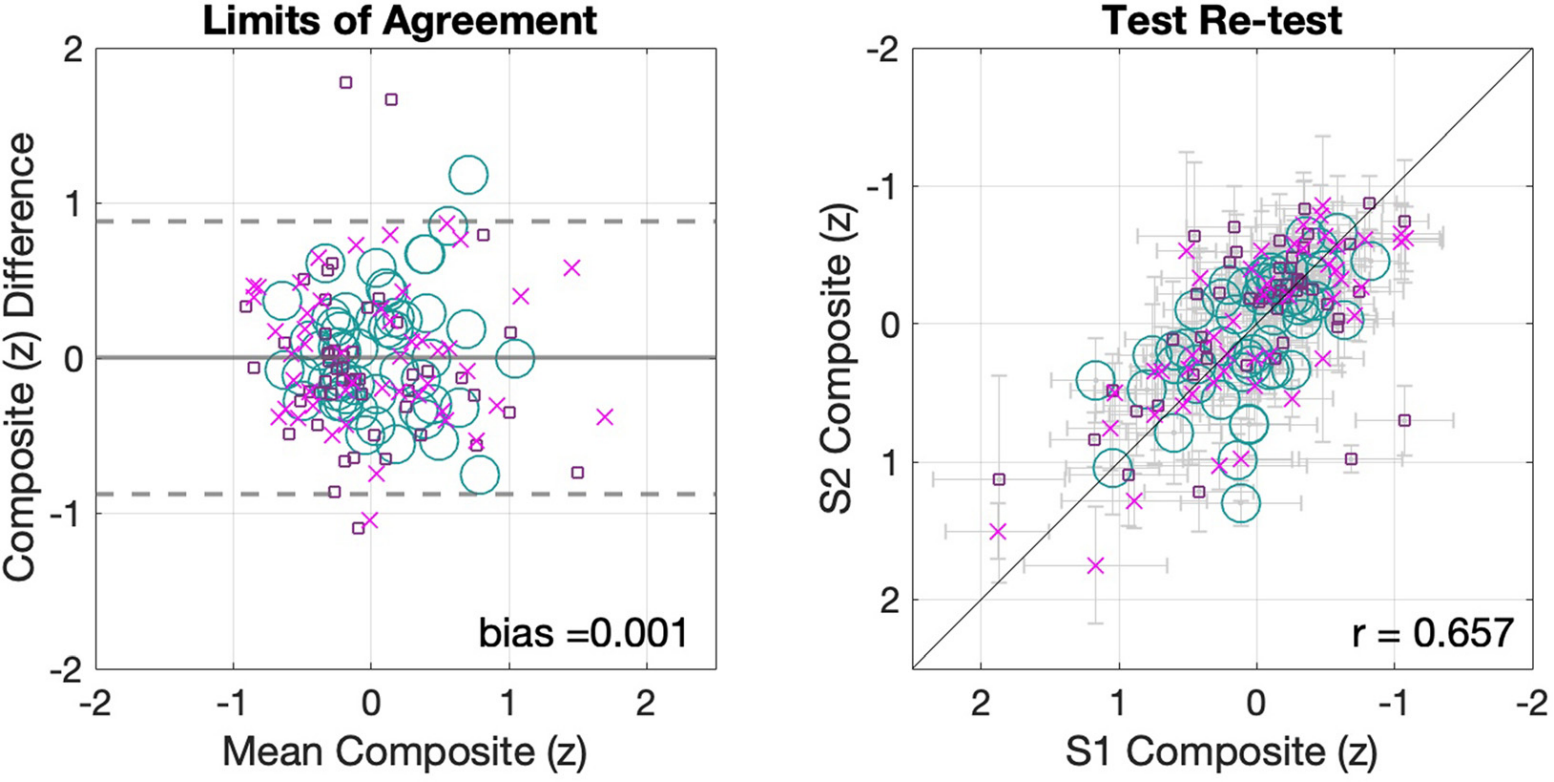
(Color online) Composite scores across all three conditions. The panel on the left shows the LoA (see Altman and Bland, 1983) for the composite scores. The panel on the right shows the scatterplot of the composite scores. The horizontal error bars indicate the SEM for session 1, whereas the vertical bars reflect the session 2 SEM.

Figure 5 shows the 95% LoA for the standardized composite scores of the whole sample across three experimental conditions (left panel). This analysis showed close to zero bias (< 0.01) and LoA of [−0.87,0.88] which indicate that 95% of repeated estimates of the composite score of the battery used in this study are expected to lie within one SD from each other in young listeners without hearing problems. In addition, Fig. 5 also shows a scatterplot of session 1 vs session 2 for the composite scores (right panel). This composite showed stronger association between sessions than each of the individual assessments (*r* = 0.65 *p <* 0.001, [95% confidence interval *=* 0.55,0.73]) and represents an alternative estimate to the LoA regarding the reliability of the battery as a whole and not of its individual assessments.

#### 1. Threshold differences across conditions

**FIG. 6.**
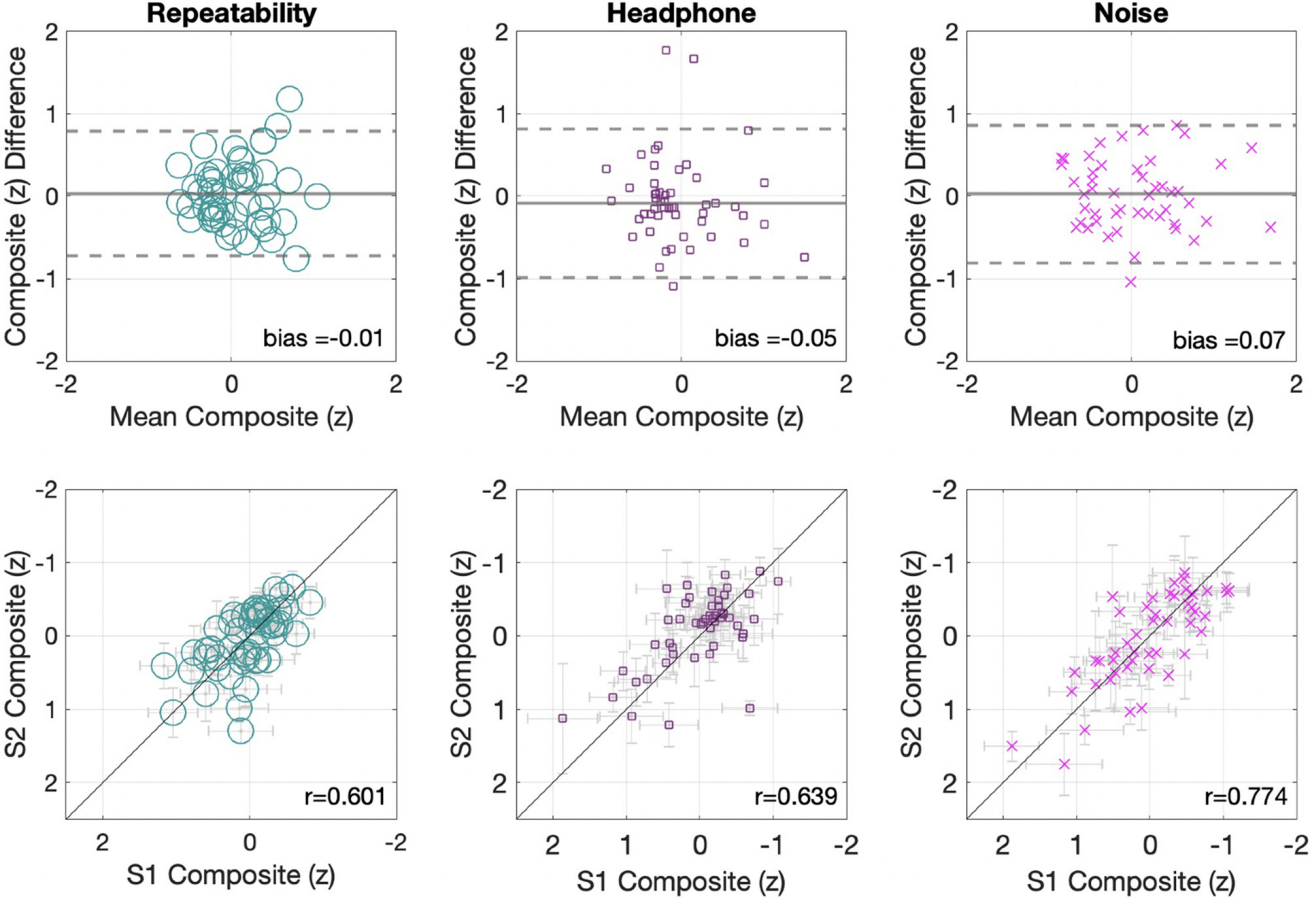
(Color online) Composite scores for each condition. The top panels show the limits of the agreement plots. The bottom panels show the composite scatterplots for each condition. The horizontal error bars indicate SEM for session 1, whereas the vertical bars reflect the session 2 SEM.

To address how composite scores changed as a function of listening condition, the composite score is plotted separately for each condition (Fig. 6). In all three experimental conditions, composite scores showed minimal bias (repeatability condition = −0.01, headphone condition = −0.05, noise condition = 0.07), LoA that resemble the aggregate sample's composite around one SD (repeatability condition [−0.72,0.79]; headphone condition [−0.98,0.81]; noise condition [−0.8,0.86]), and similar strength of association between scores of sessions 1 and 2 with (*r =* 0.601, *p <* 0.001) for the repeatability condition (standard); (*r =* 0.639, *p <* 0.001) for the headphone condition (silence); and (*r =* 0.774, *p <* 0.001) for the noise condition (noise). These correlations are within the 95% confidence intervals of the general aggregate composite *r* value.

**FIG. 7.**
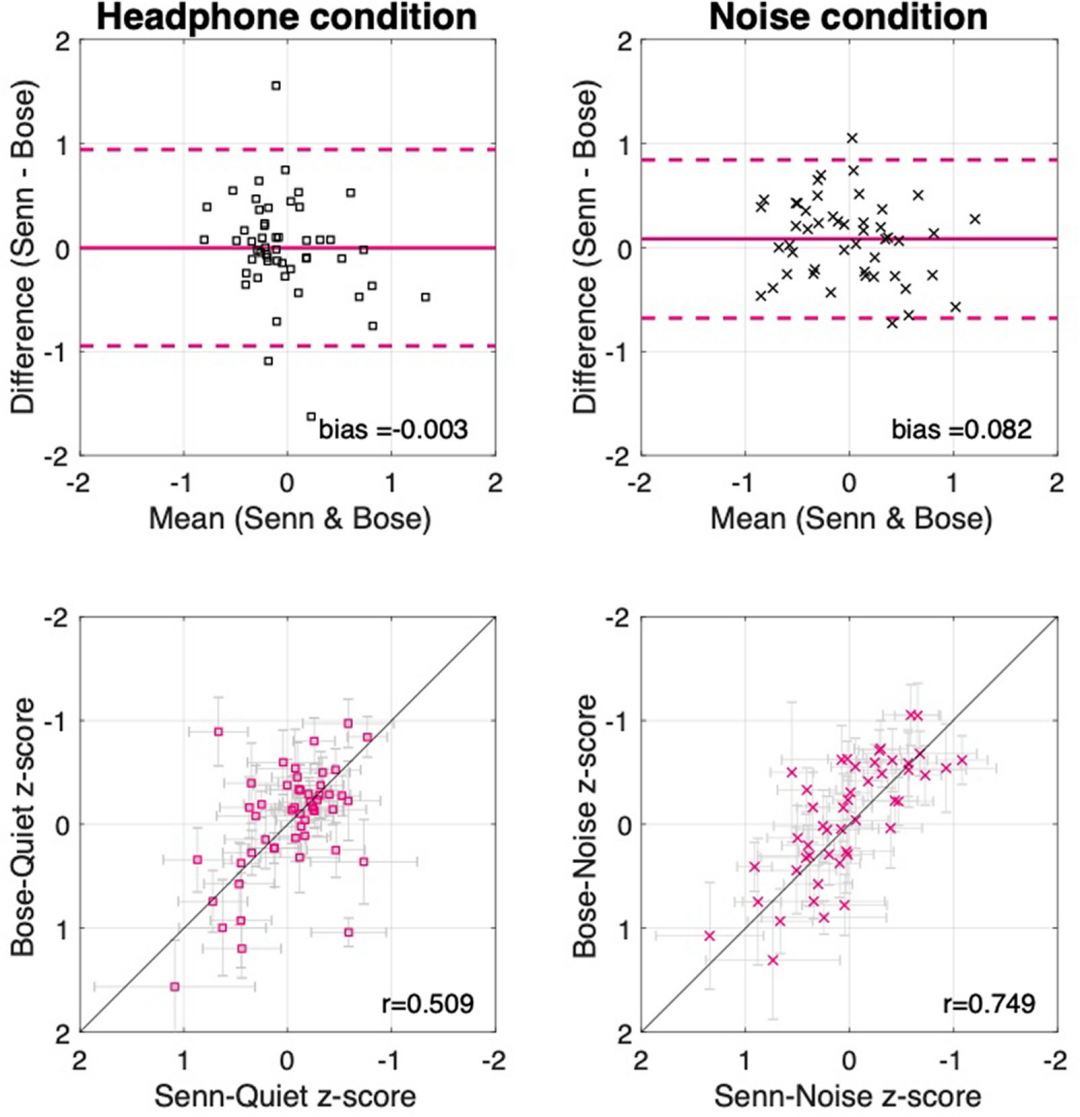
(Color online) Headphone comparison. The top panels show the LoA plots. The bottom panels show the composite scatterplots relating headphone type used. The horizontal error bars indicate the performance with Sennheiser 280 pro headphones (Sennheiser electronic GmbH and Co. KG, Wedemark, Germany) and the vertical error bars indicate performance with the Bose Quiet Comfort 35 headphones.

To formally test the hypothesis of zero bias in the threshold estimation between sessions for the different listening conditions, a series of *t*-tests was conducted. These tests failed to find significant differences in any of the conditions [repeatability condition, *t*_(50)_ = −0.34, *p =* 0.73, Cohen's *d =* −0.04; headphone condition, *t*_(50)_ = 0.3, *p =* 0.76, Cohen's *d* = 0.04); noise condition, *t*_(47)_ = −0.17, *p =* 0.86, Cohen's *d =* −0.02]. Finally, as an additional test of significance, a 3 × 2 repeated measures ANOVA with the within-subject factor session and the between-subjects factor condition was conducted to assess the overall effects of repeated measurements across a range of conditions. Again, no statistically significant effects were found for either session [*F*_(1,147)_ *=* 0.004, *p =* 0.94 *η^2^ <* 0.01] nor for condition [*F*_(2,147)_ *=* 0.92, *p =* 0.4, *η^2^ =* 0.01], and with no significant interaction [*F*_(2,147)_ *=* 0.11, *p =* 0.88, *η^2^ <* 0.01].

#### 2. Headphone comparison

To examine the effects of headphone type and the presence of environmental noise, data are presented from the headphone condition (both headphone types in silence) and noise condition (two headphone types in noise). Figure 7 shows the LoA between sessions, as well as the scatter plots that show their association, for the headphone and noise conditions separately. The data are plotted for each assessment in supplementary Figs. S2 and S3.^2^ The agreement analysis between the estimated thresholds using either set of headphones again showed minimally biased estimates [headphone condition (silence) 0.003, noise condition (noise) 0.082] and similar LoA near one SD {headphone condition (silence) [−0.94,0.94]; noise condition (noise) [−0.67,0.84} as reported in the general aggregate. Composite correlations were also similar to what is reported above with the correlation for headphone condition (silence; *r =* 0.509, *p <* 0.001) suffering due to a reduced between-subject variability. A stronger association between measures was found in the noise condition (noise) where performance between-subjects is increased in relation to the within-subject variation (*r =* 0.74, *p <* 0.001).

The stability of threshold estimates across headphones in different environmental noise conditions is a notable result as not only were the headphones different but also they shared the same output from the iPad (Apple Inc., Cupertino, CA), which was calibrated according to the Sennheiser 280 Pro (Sennheiser electronic GmbH and Co. KG, Wedemark, Germany)—and not the Bose (Bose Corporation, Framingham, MA)—headphone's mechanical output levels as detailed in Sec. II (methods). After calibration, an output level of 80 dB SPL (using the Sennheiser 280 Pro as recorded with a Brüel and Kjær Head and Torso Simulator with Artificial Ears (Brüel and Kjær Sound and Vibration Measurement A/S, Nærum, Denmark) in a VA RR and D NCRAR anechoic chamber) resulted in a level of 66 dB SPL for the Bose Quiet Comfort 35 with the high noise-cancelling setting engaged as used in all testing sessions (73 dB SPL with the noise-cancelling setting turned off). In order to allow the headphone effects to be examined without modification and avoid recalibration of the iPad between test sessions in the experiment, the settings that produced an 80 dB SPL output for the Sennheiser were used also for the Bose headphones. This meant that even in a silent environment, all of the stimuli were attenuated by 14 dB when Bose headphones were used.

Table IV shows the mean thresholds and SDs for each type of headphone in each condition and assessment. Table V shows the within-subject LoA, correlations between headphones used, and repeated measures *t*-tests that formally test differences between the estimated thresholds with each headphone for each condition and assessment separately. The data associated with these statistical tests are plotted in supplementary Figs. S2 and S3.^2^ To test for differences in threshold estimation as a function of headphone type, *t*-tests were used to compare between headphone types in each condition. Of note, since headphone type was counterbalanced across sessions, these analyses were averaged across sessions. No statistically significant effects were observed in either condition [headphone condition (silence), *t*_(50)_ *=* −0.03, *p =* 0.97, Cohen's *d =* −0.005; noise condition (noise) *t*_(47)_ *=* 1.45, *p =* 0.15, Cohen's *d =* 0.21].

**TABLE IV.** Mean thresholds and SDs for the ten assessments utilized plus the derived spatial release metric across both conditions that used different headphones. Data are presented in PART's native measurement units except for the targets-in-competition tests that have been converted to TMR. The first row of each test shows thresholds obtained using the Sennheiser 280 Pro headphones (Sennheiser electronic GmbH and Co. KG, Wedemark, Germany) and the second row of each test shows thresholds obtained using the Bose Quiet Comfort 35 headphones.

Test	Headphone *M* (SD)	Noise *M* (SD)	Units
Gap Sennheiser	2.38 (2.92)	3.18 (3.15)	Gap length (ms)
Bose	1.89 (3.29)	2.67 (3.31)	
Dichotic FM Sennheiser	0.49 (1.96)	0.48 (2.74)	Modulation depth (Hz)
Bose	0.52 (2.59)	0.49 (2.48)	
Diotic FM Sennheiser	5.91 (1.71)	5.88 (1.85)	Modulation depth (Hz)
Bose	6.26 (1.94)	5.43 (1.81)	
TM Sennheiser	1.54 (0.75)	1.32 (0.8)	Modulation depth (dB)
Bose	1.57 (0.91)	1.3 (0.9)	
SM Sennheiser	1.49 (0.72)	1.68 (0.88)	Modulation depth (dB)
Bose	1.43 (0.79)	1.66 (0.86)	
STM Sennheiser	0.94 (0.51)	0.94 (0.59)	Modulation depth (dB)
Bose	1.009 (0.62)	0.93 (0.56)	
No-notch Sennheiser	−12.25 (2.19)	−11.66 (2.1)	Target-to-masker ratio (dB)
Bose	−12.46 (2.27)	−11.66 (2.61)	
Notch Sennheiser	−32.33 (3.19)	−31.93 (2.56)	Target-to-masker ratio (dB)
Bose	−32.64 (4.83)	−32.13 (4.48)	
SR colocated Sennheiser	1.84 (2.22)	1.87 (2.46)	Target-to-masker ratio (dB)
Bose	2.07 (1.3)	1.39 (3.04)	
SR separated Sennheiser	−4.82 (3.25)	−3.51 (4.04)	Target-to-masker ratio (dB)
Bose	−4.27 (2.59)	−4.25 (4.05)	
Spatial release Sennheiser	6.66 (3.05)	4.47 (3.54)	SR (Separated - colocated) (dB)
Bose	6.35 (2.8)	4.77 (3.59)	

**TABLE V.** LoA and significance testing for the ten assessments comparing headphones used in two conditions. The first row shows the headphone condition and the second row shows the noise condition. Positive values on the bias column indicate better performance when using the Sennheiser headphones (Sennheiser electronic GmbH and Co. KG, Wedemark, Germany) except on the spatial release metric, which is the only scale in which larger magnitudes indicate better performance. “*” indicates significance at α = 0.05.

Test	Bias	LoA	Units	*r* (*p*)	*t* (*p*)	Cohen's *d*	df
Gap (noise)	−0.33	[−3.63 to 2.97]	log 2 (ms)	0.47 (<0.01)*	1.37 (0.17)	−0.2	48
	−0.25	[−3.36 to 1.67]		0.56 (<0.01)*	1.07 (0.28)	−0.14	45
Dichotic FM (noise)	0.07	[−2.62 to 2.77]	log 2 (Hz)	0.35 (0.01)*	−0.37 (0.7)	0.06	50
	0.02	[−2.21 to 2.25]		0.66 (<0.01)*	−0.12 (0.9)	0.01	47
Diotic FM (noise)	0.08	[−1.88 to 2.05]	log 2 (Hz)	0.34 (0.01)*	−0.58 (0.56)	0.96	47
	−0.11	[−1.72 to 1.49]		0.56 (<0.01)*	0.94 (0.34)	−0.12	46
TM (noise)	0.03	[−1.58 to 1.64]	*M*	0.53 (<0.01)*	−0.28 (0.77)	0.03	50
	−0.01	[−1.7 to 1.67]	(dB)	0.49 (<0.01)*	0.13 (0.89)	−0.02	44
SM (noise)	−0.06	[−1.55 to 1.43]	*M* (dB)	0.5 (<0.01)*	0.55 (0.58)	−0.08	46
	−0.01	[−1.78 to 1.75]		0.47 (<0.01)*	0.11 (0.9)	−0.01	43
STM (noise)	0.06	[−1.19 to 1.32]	*M* (dB)	0.38 (<0.01)*	−0.71 (0.47)	0.11	47
	−0.004	[−1.45 to 1.45]		0.19 (0.23)	0.03 (0.97)	−0.007	39
No-notch (noise)	−0.2	[−5.02, 4.6]	TMR (dB)	0.39 (<0.01)*	0.59 (0.55)	−0.09	49
	0	[−6.1 to 6.1]		0.14 (0.34)	0 (1)	0	43
Notch (noise)	−0.31	[−8.72 to 8.09]	TMR (dB)	0.49 (<0.01)*	0.51 (0.6)	−0.07	49
	−0.2	[−9.52 to 9.11]		0.17 (0.25)	0.27 (0.78)	−0.05	41
Co-located (noise)	0.23	[−3.77 to 4.24]	TMR (dB)	0.42 (<0.01)*	−0.82 (0.41)	0.12	50
	−0.48	[−5.89 to 4.91]		0.51 (<0.01)*	1.13 (0.26)	−0.17	40
Separated (noise)	0.54	[−6.31 to 7.4]	TMR (dB)	0.29 (0.03)*	−1.12 (0.26)	0.18	50
	−0.74	[−6.32 to 4.83]		0.75 (<0.01)*	1.79 (0.07)	−0.18	46
SpatialR (noise)	−0.31	[−8 to 7.37]	TMR (dB)	0.107 (0.45)	0.57 (0.57)	−0.1	50
	0.3	[−6.28 to 6.88]		0.55 (<0.01)*	−0.56 (0.57)	0.08	39

As an additional test of significance, a 2 × 2 repeated measures ANOVA with the within-subject factor headphone and the between-subjects factor condition was conducted to assess headphone effects across experimental conditions. Again, no statistically significant effects were found for neither headphone [*F*_(1,97)_ *=* 0.8, *p =* 0.37, *η*^2^ *<* 0.01] nor for condition [*F*_(1,97)_ *=* 0.01, *p =* 0.91, *η*^2^ *<* 0.01], and with no significant interaction [*F*_(1,97)_ *=* 0.9, *p =* 0.34, *η*^2^ *<* 0.01].

In summary, the data failed to show any systematic effect of headphone type when participants were tested in either silent or noisy environments. These composite analyses further support the reliability of PART and suggest that it may be achieved with or without active noise cancelling technology and in the presence of moderate environmental noise. These results also suggest that even a 14 dB difference at the mechanical output level did not produce noticeable differences in performances for these undergraduate students with hearing in the normal range.

## IV. DISCUSSION

This study examined the validity and reliability of a battery of ten assessments that evaluate different aspects of the central auditory function using the PART application applied to young adult listeners without reported hearing problems. Overall, results show that thresholds can be obtained that are highly consistent across sessions and very similar to those reported in laboratory settings obtained with more traditional equipment and, in some cases, with extended testing and training (see Table III). Furthermore, results from the repeatability condition were replicated in the headphone and noise conditions, demonstrating that PART produces consistent threshold estimates across a variety of settings and equipment. Overall, these results suggest that the PART platform can provide valid measurements across a range of listening conditions.

An important utility for this study is that it provides an initial normative dataset for young adult listeners for the PART tasks reported and eventually as a reference for patient populations. However, substantial work is required before PART will be appropriate for clinical use. For example, while the tasks included in this first battery were chosen based upon prior literature, suggesting possible sensitivity in understanding listening disorders, these data do not capture variations in age and do not include effects of differences in the hearing threshold (see Jakien and Gallun, 2018). Future work will involve developing a similar normative dataset for this battery. With such a dataset, it would be possible to determine which combinations of tests are most sensitive in distinguishing between different disorders. Likewise, the reliability of measures needs to be established in different populations that may have more difficulty with the procedures than the college student population reported here.

Further, although learning effects are among the smallest effects observed in this dataset, they must be explored in relevant patient populations and potentially accounted for when interpreting test results. In both cases, our results suggest that either repeated measures or adjustments to adaptive procedures will be required to increase reliability in patient populations. Still, the fact that thresholds similar to those found in the literature can be obtained on a large number of tests within a short period of time, using consumer-grade technology provides optimism that PART will be useful in the clinic.

The criteria we used for outlier rejection was justified by the goal of creating a normative dataset, but it is important to note that the field holds a variety of different views regarding outlier rejection. The current choice is simply definitional in that “normative” refers to a normal distribution and, thus, it is appropriate to reject data falling far outside of this distribution. Nevertheless, there was a minimal effect on population estimates of means and SDs whether or not outliers are excluded (see supplementary Table I^2^). The supplementary Fig. 1,^2^ which shows the data with the outliers circled, reveals that the main impact of including outliers is to make it more difficult to see the normal range of the dataset. Another important question is whether or not it is possible to say something meaningful about which listeners gave data that was then rejected. For example, are they impaired in auditory processing or do they represent a typical variation of the larger population? It seems unlikely that these participants had any significant hearing loss as they all self-reported to have no hearing difficulties, which is considered a reasonable indication for normal hearing (Vermiglio *et al.*, 2018) and were able to detect a 45 dB SPL 2 kHz pure tone, which assured audibility minimum criteria. Moreover, as indicated in supplementary Table I, most outlying cases were not consistent across sessions, and as can be seen in supplementary Fig. S1 are within the normal range in one of the testing sessions.^2^ This suggests that many of the outliers were either inattentive, unmotivated, confused, or otherwise noncompliant in one of the sessions. Further, some of the outliers were actually in the supra-normal hearing range, again evidence against outliers being indicative of hearing impairment. Still, while it is reasonable to suggest that outliers do not represent normative or dominantly systematic effects, they are still a concern and do need to be considered when contemplating clinical implications especially of a single test. In particular, it is important to keep in mind the expected probability (ranging from 1% to 8%, depending on the test) of getting an unreliable test result when using these tests on this platform.

Another issue of concern is the extent to which performance may change systematically across testing sessions. LoA plots, correlational analyses, and statistical tests of session effects all provide complementary information on changes in performance over time. In general, systematic bias of threshold estimates across sessions was minimal and similar to the bias observed across levels of performance (see LoA plots). The expected differences among measures across sessions are estimated in the LoA between sessions. While significance testing revealed some differences between sessions in some of the tests, the effect sizes we found are small and typically comparable to the smallest step sizes used in the procedures. Further, these can now be considered as test-retest effects in future work. Consistent with this, reliability was further quantified using the Pearson *r*, which is not sensitive to systematic effects of the testing session. Although correlation is highly reactive to small changes in between-subject variability (see supplementary Table ST1^2^) and cases with reduced between-subject variability, it presents complementary information regarding the relation among within-subject and between-subject variabilities. When the assessments with smaller *r* values in complement to the LoA plots are examined, it can be seen that in most cases the LoA closely resemble other tests with higher *r* values. This is an indication that *r* is decreasing due to restricted ranges of good performance as was to be expected in this sample of normal listeners.

It is notable that thresholds were consistent across different external noise conditions (repeatability and headphone vs noise conditions) and across different types of headphones (repeatability vs headphone and noise conditions; see Figs. 6 and 7). Also of note, the correlations were higher for the condition with external cafeteria noise without an increase on the LoA. This is important because a test platform that is portable, automatic, and rapid can only be successfully exploited if it is able to provide accurate measurements that can be collected in a variety of potentially less than optimal settings. Here, we have shown that PART was able to obtain estimates of central auditory function that resemble those found under laboratory conditions despite using untrained listeners tested in settings that resemble a typical, moderately noisy, university cafeteria. PART should be considered as a supplementary tool in the clinic that can be used to collect valuable information about a person's hearing capabilities with little need for supervision from a clinician. These results also suggest that this system and these tests are robust to the presence of moderate noise and substantial variability in sound output levels.

This study also compared the use of headphones with an active noise-cancelling technology to those with passive attenuation. We considered it worthwhile to test this technology because it is now widely available, but little is known about the advantages and disadvantages it could represent for auditory testing. We failed to find a statistically significant effect between threshold estimates obtained for the different headphones under both silent and noisy listening conditions. In other words, estimated thresholds were similar for the Sennheiser 280 Pro (Sennheiser electronic GmbH and Co. KG, Wedemark, Germany) in silence (headphone condition) and this lack of difference manifested similarly in noisy conditions (noise condition). This suggests that the passive attenuation provided by the Sennheiser 280 Pro is sufficient to obtain reliable measurements in less than optimal external noise conditions outside of the sound booth. Also, it suggests that the differences between the headphones, including the active noise-cancelling algorithm, are not changing the signal in any way that results in significant reductions in performance. Perhaps the noise-cancelling signal processing was inactive or operating at low frequencies that did not affect performance. In any case, threshold estimation held constant across the headphone technologies used with a single calibration profile (the same output from the iPad; Apple Inc., Cupertino, CA). These data serve as verification that relatively inexpensive auditory hardware can be used to test auditory function in a variety of settings with sufficient precision to provide clinical evidence of central auditory function in individual listeners.

PART can, thus, appropriately be considered as a valid platform for testing several aspects of central auditory processing. It is robust to moderate levels of ambient noise and small variants in equipment and procedure. The reported data can now be used as a normative baseline against which auditory dysfunction can be identified in future work. However, clinical research will be needed to determine how thresholds vary as a function of age and different degrees of hearing loss. The reliability analysis reported here applies only to young listeners with normal hearing. Future work will need to address whether threshold estimates from PART can be reliably obtained for older listeners with varying degrees of hearing loss and to determine the extent to which the measured reliability in this work is adequate for identifying a central auditory processing deficit. This next step is feasible considering that the PART platform is highly accessible given its relatively low cost in terms of expense (it only requires a computer tablet and headphones), time (the whole battery of ten assessments takes less than one hour), human resources (it runs the assessments automatically, one after another, including instructions and breaks), and it can be used in a range of environmental settings suitable for testing [from the anechoic chamber as in Gallun *et al.* (2018) to noisy cafeteria conditions]. Thus, PART has the potential to provide a supplementary tool to gather the quantity and variety of psychophysical measures of auditory function that will allow us to translate laboratory findings into the clinic to inform clinical practice.

## Footnotes

^1^ See https://braingamecenter.ucr.edu (Last viewed September 24, 2020).

^2^ See supplementary material at https://doi.org/10.1121/10.0002108 for further analysis on this dataset.

^3^ See https://www.cdc.gov/niosh/topics/noise/app.html (Last viewed September 24, 2020).
